# Medical and Surgical Cases Occurring in the Sisters of Charity Hospital

**Published:** 1869-10

**Authors:** W. W. Miner

**Affiliations:** Member of the Class


BUFFALO

Medical and Surgical Journal.

VOL. IX. OCTOBER, 1869. No. 3.

### Original Communications.

### ART. I.—*Medical and Surgical Cases occurring in the Sisters of Charity Hospital.*

Reported by W. W. MINER, A. B , Member of the Class.

PROF. ROCHESTER'S MEDICAL CLINIC:

*Case I.*—Mr. M ——, aged thirty-three, just entered the ward, has cerebral disturbance, considerable redness of tongue and tonsils, and also a scarlet fever eruption on his body, while a white diphtheritic exudation appears on the pharynx. The symptoms of diphtheria and scarlet fever are similar, as also their sequelæ. Some physicians affirm that diphtheria and scarlet fever are different phases of the same disorder, but I have seen diphtheria follow small pox as well as scarlet fever, and believe that this case is one of scarlet fever, indicated by redness of tongue and tonsils, and by the eruptions, while a little diphtheritic exudation still remains in the pharynx. Quinine and a nutritive diet have been given, and the patient is convalescing. Scarlet fever is a zymotic affection, periodically prevalent in its appearance, of a limited duration, and is communicable. Prophylactic efforts are on the safe side at least, and are rightly made use of. As evidence, allow me to cite an instance which came under my observation. In a place one hundred miles distant, a child died of scarlet fever, the nurse of the child, who had been very close in her attentions during its illness, came to this city and entered a family having an only child, ten years of age. With negligence which seems almost criminal, she wore the same thick woolen dress that she had worn while attending the scarlet fever patient. Two weeks after she entered this family, the child died of a severe form of scarlet fever. No other cases occurred in the neighborhood. One fact of this kind is worth more than ten negative facts. I, myself, have been exposed to scarlet fever a thousand times, perhaps, and have never had the disease, still I believe it to be communicable. The treatment is hygienic: probably the duration of the disease is not shortened by medical treatment. Dr. West, of England, and Hughes Bennett, employ a bath, whereby the sequelæ which may occur are avoided, or are rendered milder in form. Scarlatina anginosa requires local treatment, and that early. A gargle, medicated paper for fumigating, and a camel's hair pencil, are means of applying remedies. Dover's powders may be given, if there is considerable weakness; and quinine prescribed, if there is delirium or prostration. Emetics used to be employed; but they should never be given in the early stages of the disease, as vomiting and diarrhœa are often among its early indications; but, in the last stages of the affection, an emetic sometimes remove an accumulation, and relieves a sense of oppression in the throat and fauces. Antiseptics are often advantageously employed: mineral acids are used for this purpose, and also a combination of chlorate of potassa, and dilute hydrochloric acid, which liberates chlorine; carbolic acid is a new agent, and is well spoken of; the crystallized form should always be used; and one-fourth of a grain is the dose for a child, while one-half to one grain may be given an adult, but if vomiting or irritation ensue its use must be stopped. Belladonna, which has been extensively employed, both as a remedial agent and a prophylactic, has no good effects, and its use is deleterious rather than otherwise. Bathing constitutes a most excellent means of treatment, but may not always be practicable, and do not order it in a hovel where the proper care cannot be taken; but rather anoint the body with fresh lard, which will allay itching and abate the fever. Five drops of dilute hydrochloric acid may be given once in four hours as an antiseptic, and a gargle used after the following formula:

℞
Carbolic Acid, (crystals,) - - - ʒss.
Glycerine, - - - - - ℥ii.
Saturated Sol. Potass. Chlorat., - - ℥iii.
Tinct. Myrrh, - - - - ℥j.
M.

Do not medicate too much, but rather give as little medicine as your reputations as physicians will allow.

I wish to caution you against one of the sequelæ of this disease, which is very easily controlled in its early stages, but which becomes a grave disorder if it is not anticipated and checked. This is albuminuria, in which affection the kidneys suffer from sanguinolent engorgement, whereby the functions of that organ are seriously impeded, and uræmia rendered likely to occur. Albuminuria follows mild cases of scarlet fever as well as severe ones, and may be detected by the appearance in the urine, of the cylindrical fibrinous deposits of the kidneys, together with bits of epithelium from the urinary ducts, and the presence in it of albumen, which is rendered visible on the addition of nitric acid, or by the application of heat. The patients often have a swollen appearance of the upper extemities after having arisen from bed, which shortly gravitates to the lower extremities, and often results in general anasarca. The urine of a scarlet fever patient should be examined repeatedly in order to detect this disease, which, if occurring, may be treated with saline aperients, diaphoretics, or with a warm bath once or twice daily; but specific medication must be employed in the use of tannin or gallic acid, of which the former is more prompt in its action. Three grains of tannin, dissolved in one drachm of glycerine, are to be given once in four hours. Tannin will generally increase the quantity of the urine, and the albumen will disappear at the same time; its action is said to be mechanical, in forcing out the fibrinous casts from the uriniferous ducts; its use should not be continued for more than three days unless improvement follows. It may be succeeded by the giving of muriated tincture, or persulphate of iron, in doses of two or three drops, once in three hours. Cupping may be resorted to, but blistering should be avoided. Among the sequelæ of this disease, we notice effusions on the brain, bronchitis, pneumonitis, synovitis, pleuritis, endocarditis, chorea, and Bright's disease, which is a fatty or granular degeneration of the kidneys.

Dr. McNutt, of Missouri, claims a specific for albuminuria in an infusion of the inner bark of elder in cider, the virtue of which is yet to be established. A careful attention to the urine is urged in all cases, the importance of which is shown by a case, to which I was called, of a small child who had convulsions, followed by coma, and who was said by its parents to have been perfectly well until the appearance of these alarming indications. On inquiry, however, I found that, five or six days before, there had been a little scarlet rash seen on the child, which did not so much as hinder the child from its play, but was an explanation of the severe albuminuria and uremia from which the child barely recovered.

*Case II.*—J—— N——, aged forty-four, a-long-shore man; been complaining three months, has a cough and grows weak, says he spits bright red blood on arising from bed in the morning. Pulse eighty-four, somewhat anæmic in appearance, has a sore leg; his father had a leg similarly affected, which trouble he calls rheumatic; it has the mark of an old ulcer, which he says troubled him six months. Spots of discoloration, scattered over his body, point to a specific cause, which may, however, have been hereditary; but Dr. Hamilton says that copper colored spots are often an accompaniment of chronic ulcers of no specific origin. These ulcers should be made to heal very slowly while the general health is being recruited, otherwise the liver, lungs, or other organs may become affected. The patient's apparent hæmoptysis may be occasioned by blood coming from the posterior nares. We will lay back the clothing and examine the chest, first as to its symmetrical developement; we notice there is more depression in the right infra-clavicular region than in the left. On the back we see the marks of previous cupping, which the patient says was donewhile he was in the army. Next we will auscultate, which is better done before percussing, and sometimes obviates the necessity of percussion. We find better respiratory action in the right lung than in the left, hence the preliminary observation might have misled us. The respiratory murmur of the left lung is not good, and the sound of percussion on that side is a little duller than that on the right. The patient is hoarse and has a cachetic appearance, and we diagnose the case as imperfectly healed pneumonia. Have seen sanguinolent expectoration in such cases. A tonic treatment is ordered, consisting of quinine, iron and iodide of potassa.

*Case III.*—I wish to recall to your minds a case which was present-you at the last clinic, which seems worthy of notice on account of its abrupt termination. The bodies of six of the dorsal vertebræ of the patient had undergone softening, and great tenderness was felt on percussion of the dorsal region. Cod liver oil, a tablespoonful; iron, one grain; and strychnia, one-twentieth of a grain, were the remedial agents which had been employed. The patient was suffering from pain in his muscles, and one-fourth grain of morphine was ordered to be given hypodermically when necessary to relieve him. The nurse undertook one evening to give one-half grain morphia hypodermically, but was unsuccessful in introducing more than half that quantity. The patient sent again for a second administration of the anodyne, but the nurse refused to give a second injection. At half-past nine in the evening the patient was asleep, at half-past eleven he conversed with patients near him, at half-past two he was sleeping all right, and between five and six in the morning he was found dead in his bed, no premonition or evidence of such occurrence having been apprehended. A post-mortem examination was avoided by the friends who took away the deceased before the medical officers were consulted, but I suspect that death was occasioned by the rupture of an aneurism of the descending aorta. The patient had a care-worn look, and suffered severe and continued pain in the back. Caries of the bodies of the vertebrae was produced probably by the pressure of the aneurism upon them. This case well introduces a very interesting specimen of a heart which, through the kindness of Drs. Potter and Taylor, I am able to present you. The patient came to me nine months since to inquire if he had got to die. There was entire absence of the radial pulse, and but a slight temporal one. An examination of the præcordial region showed that the first and second sounds of the heart were normal. The sternal region gave a purring, smart aneurismal thrill. Latterly the patient has had temporary paralysis from the lack of supply of blood to the right hemisphere of the brain. There was no difficulty in the diagnosis, and this specimen very excellently presents the peculiarities of the case, which are complete obstruction by fibrinous clots of the right subclavian and carotid arteries, with partial obstruction of the subclavian and aphthous deposit lining the aorta. The whole arch of the aorta is involved in an aneurism, which is found to have produced caries of the vertebræ adjoining it. The heart is of normal size, and the valves are perfect, but a patulous and enlarged condition of the left common carotid artery exists, which may have tended to lessen the aneurismal indication in the heart's action.

PROF. EASTMAN'S SURGICAL CLINIC:

*Case IV.*—Stephen ——, aged fifteen, has stricture of the urethra. Strictures of the urethra are of three kinds—spasmodic, congestive, and organic: of these the first and second are of limited duration, but sometimes change into the third, or organic form, in which the contraction of the urinary canal is permanently established. Permanent stricture results from injury to the perineal region, or more frequently succeeds repeated attacks of gonorrheal affections. In either case the inflammation which exists in the tissues about the urethra cause an effusion of plastic lymph, whereby the mucous membrane of the urethra is thickened, so that while its external diameter is increased, the internal urinary passage is diminished or entirely obliterated. The patient before you, received an injury producing laceration of the perineum, which has resulted in stricture. He has been under the charge of two of the city physicians, and now he has come to the hospital. Efforts to introduce a catheter have been unavailing, and the urine no longer flows, but comes away drop by drop. By percussion we determine that the bladder is not distended; and, by flexing the thighs, the tension of the abdominal muscles is relaxed. We make use of a steel sound, which we are at present able to introduce a little way into the stricture, as is indicated by the firmness with which the sound is embraced, and with this we expect ultimately to effect an entrance to the bladder. It is well to divert the attention of the patient while introducing a catheter, and thus avoid the spasmodic contraction of the muscular fibres of the urethra. Very slight pressure should be used in introducing the sound, else inflammation may be excited, which would at least delay our efforts at relief, and might cause still greater constriction of the urinary canal. Beyond the stricture, the urethra frequently becomes enlarged so as to form a *cul de sac*; and a former patient had a calculus two centimetres in diameter, removed by lithotomy from a sac thus formed. A patient under treatment, in the General Hospital, for the removal of stricture, is now able to wear a catheter one hour in the morning, and another hour in the evening; and it is also expected, in the present case, that by the stimulus of continued mechanical pressure, the adventitious deposits will be absorbed, and by continued dilatation, the normal size of the urinary canal will be established.

